# Supplementary material for: Ablation of CD8+ T cell recognition of an immunodominant epitope in SARS-CoV-2 Omicron variants BA.1, BA.2 and BA.3
Source: Nat Commun. 2022 Oct 27;13:6387. doi: 10.1038/s41467-022-34180-1 (PMC9607807; doi:10.1038/s41467-022-34180-1)
Supplement: Supplementary file 1 — Supplementary Information [file 41467_2022_34180_MOESM1_ESM.pdf]

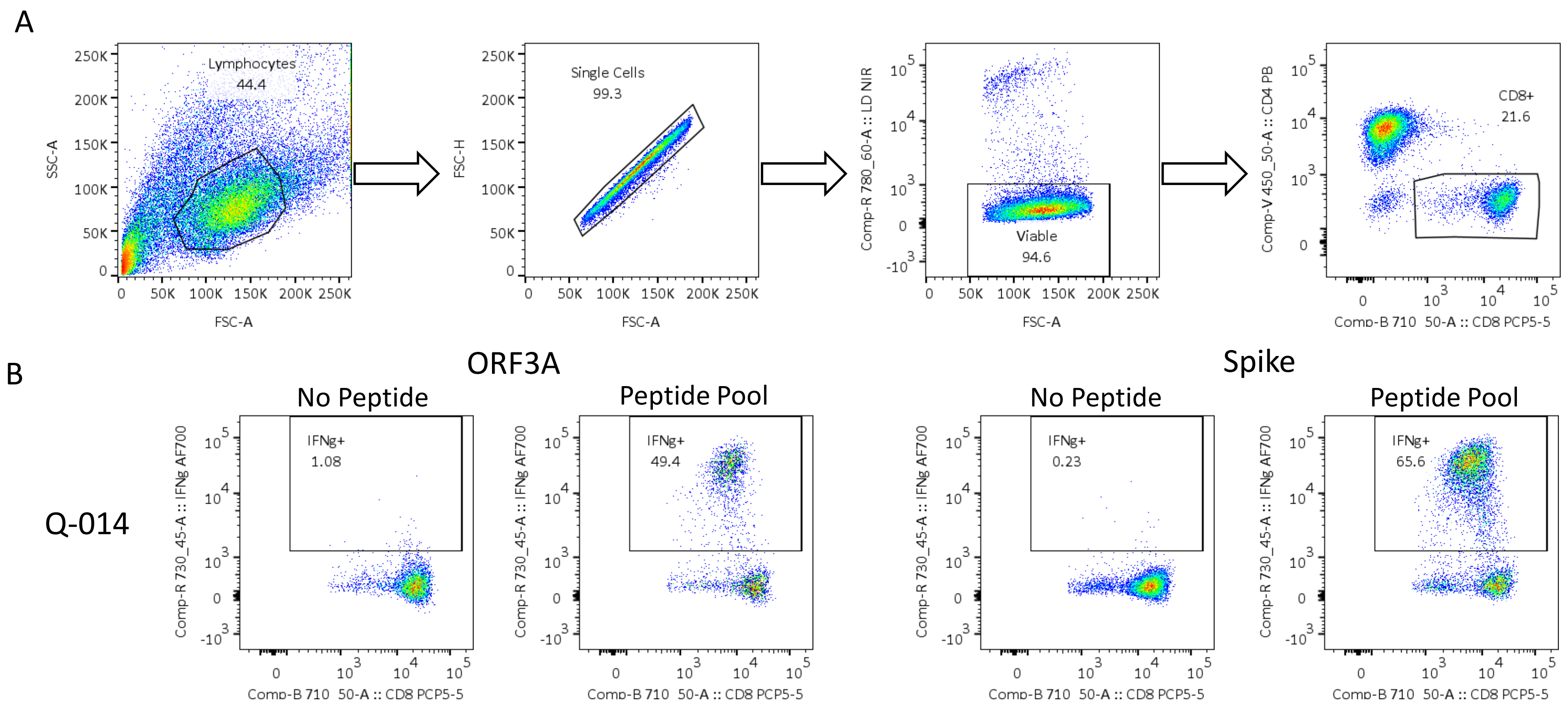

Supplementary Figure 1: (A) Representative gating strategy used to identify CD8⁺ T cells **(B)** Representative IFN-γ production by ORF3A and spike-specific CD8⁺ T cells from a COVID-19-convalescent HLAA*29:02-positive participant.

A

Pool (% IFN-γ^+)	1 (0.01)	2 (0.00)	3 (0.08)	4 (0.00)	5 (40.89)	6 (48.79)	7 (0.14)	8 (0.00)	9 (0.05)	10 (0.00)	11 (0.09)	12 (1.55)	13 (1.87)
**14 (0.00)**	1	2	3	4	5	6	7	8	9	10	11	12	13
**15 (0.00)**	14	15	16	17	18	19	20	21	22	23	24	25	26
**16 (0.00)**	27	28	29	30	31	32	33	34	35	36	37	38	39
**17 (0.00)**	40	41	42	43	44	45	46	47	48	49	50	51	52
**18 (0.09)**	53	54	55	56	57	58	59	60	61	62	63	64	65
**19 (0.00)**	66	67	68	69	70	71	72	73	74	75	76	77	78
**20 (2.82)**	79	80	81	82	83	84	85	86	87	88	89	90	91
**21 (0.01)**	92	93	94	95	96	97	98	99	100	101	102	103	104
**22 (0.00)**	105	106	107	108	109	110	111	112	113	114	115	116	117
**23 (48.99)**	118	119	120	121	122	123	124	125	126	127	128	129	130
**24 (0.00)**	131	132	133	134	135	136	137	138	139	140	141	142	143
**25 (0.00)**	144	145	146	147	148	149	150	151	152	153	154	155	156
**26 (0.09)**	157	158											

B

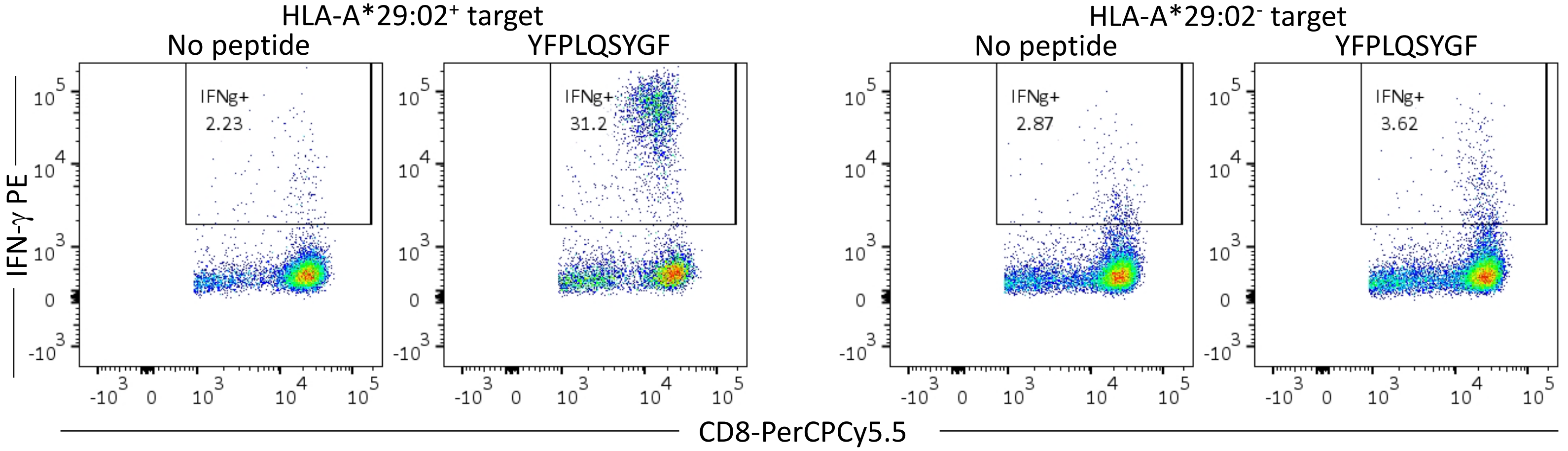

C

Pool (% IFN-γ^+)	1 (0.11)	2 (0.16)	3 (0.27)	4 (3.82)	5 (2.57)	6 (0.68)	7 (15.38)	8 (14.98)
**9 (0.22)**	1	2	3	4	5	6	7	8
**10 (0.08)**	9	10	11	12	13	14	15	16
**11 (1.25)**	17	18	19	20	21	22	23	24
**12 (0.44)**	25	26	27	28	29	30	31	32
**13 (4.07)**	33	34	35	36	37	38	39	40
**14 (0.23)**	41	42	43	44	45	46	47	48
**15 (11.08)**	49	50	51	52	53	54	55	56
**16 (1.28)**	57	58	59	60	61	62	63	64
**17 (0.15)**	65	66	67	68	69	70	71	

Supplementary Figure 2: (A) Spike (S-1) matrix peptide pool responses for Q-031 T cells. Matrix pool numbers are highlighted in bold. Values in parentheses represent the percentage of IFN-γ^+ CD8$^+$ T cells following subtraction of no peptide control. Numbers in red represent the dominant reactive peptides (122 - GFNCYFPLQSYGFQP; 123 - YFPLQSYGFQPTNGV). **(B)** HLA restriction of YFPLQSYGF-specific T cells from Q-031. **(C)** ORF3A matrix peptide pool responses for Q-031 T cells. Matrix pool numbers are highlighted in bold. Values in parentheses represent the percentage of IFN-γ^+ CD8$^+$ T cells following subtraction of no peptide control. Numbers in red represent the dominant reactive peptides (55 - VVLHSYFTSDYYQLY; 56 -SYFTSDYYQLYSTQL).

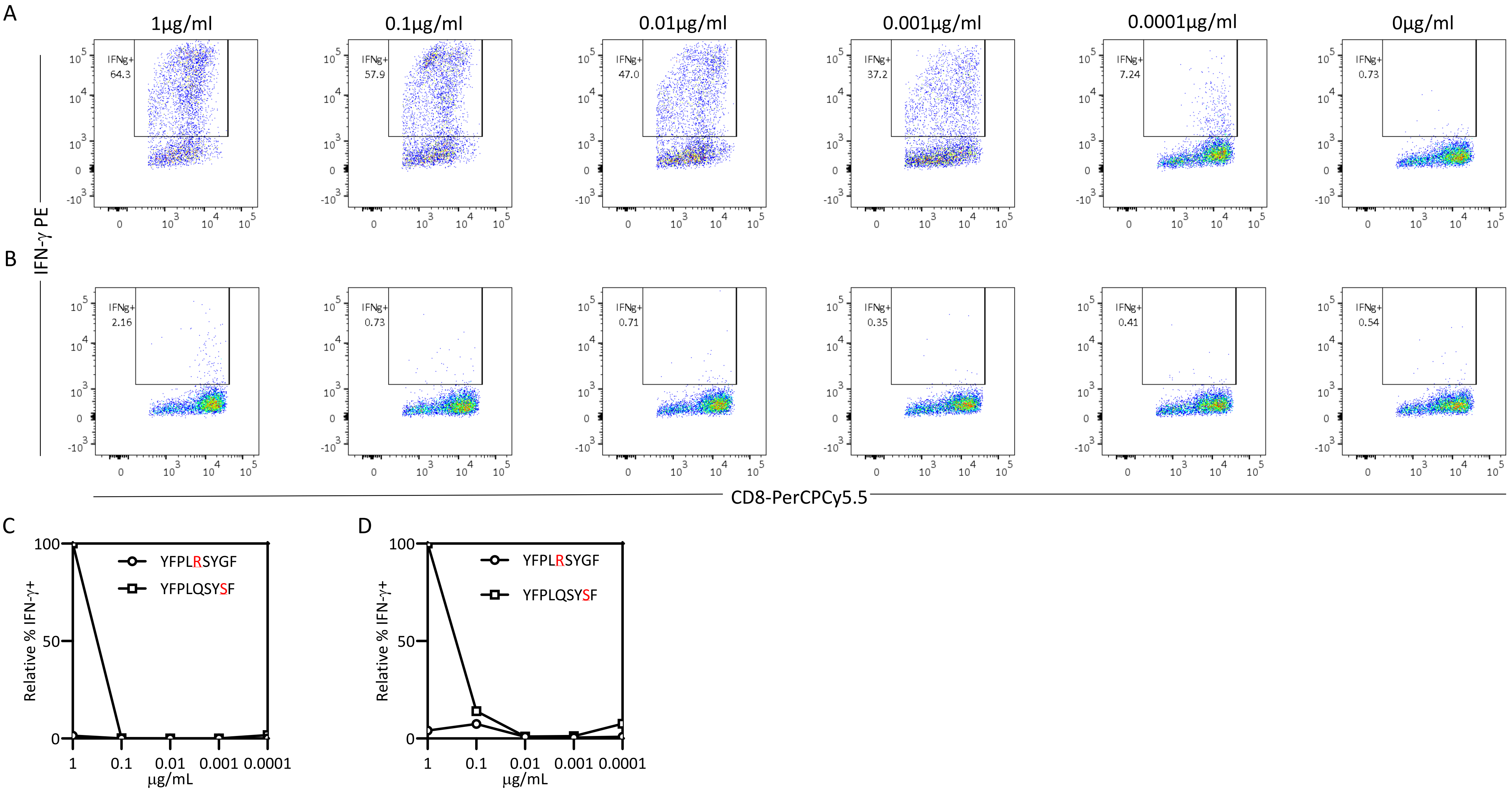

Supplementary Figure 3: Representative intracellular cytokine analysis following stimulation of Q-031 T cells with serial dilutions of **(A)** YFPLQSYGF and **(B)**YFPLRSYSF. Functional avidity analysis was performed to assess the impact of single amino acid substitution at position 5, Q to R and position 8, G-S, on recognition by T cells from **(C)** Q-031 and **(D)** Q-043 .

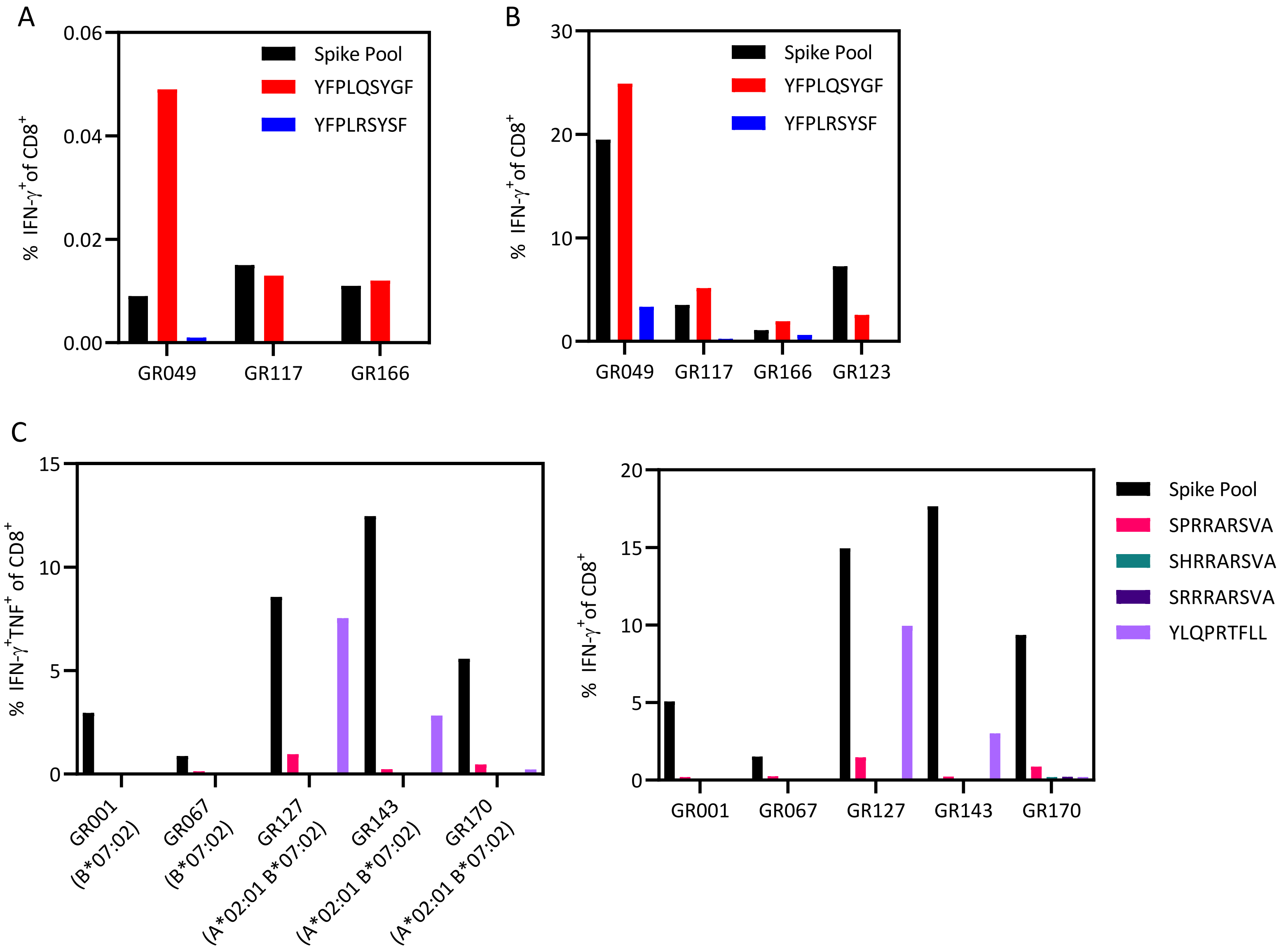

Supplementary Figure 4: IFN-γ response to spike peptide pool, YFPLQSYGF and YFPLRSYSF in PBMC **(A)** and following in vitro expansion **(B).** Both IFN-γ^{+}TNF^{+} and IFN-γ^{+} response was assessed to spike peptide pool, SPRRARSVA, SHRRARSVA, SRRRARSVA and YLQPRTFLL following in vitro expansion in HLA-B*07:02 volunteers.

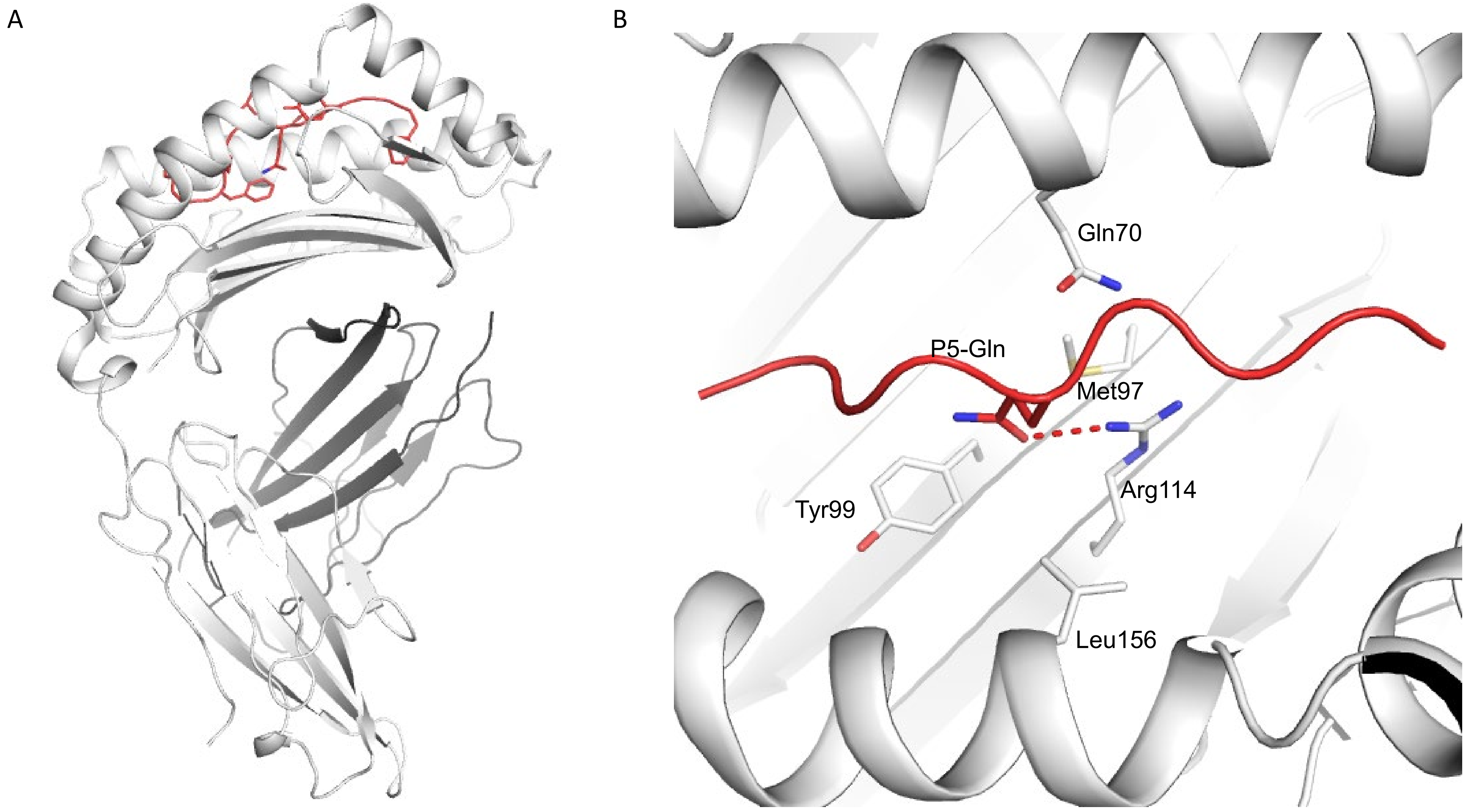

Supplementary Figure 5. Crystal structure of HLA-A*29:02-YFP complex. **(A)** Overall structure of the HLA-A*29:02, with the heavy chain as white cartoon and the β2m as grey cartoon, in complex with the YFP peptide in red cartoon and stick. **(B)** Interaction of the secondary anchor residue P5-Gln from the YFP peptide (red cartoon)

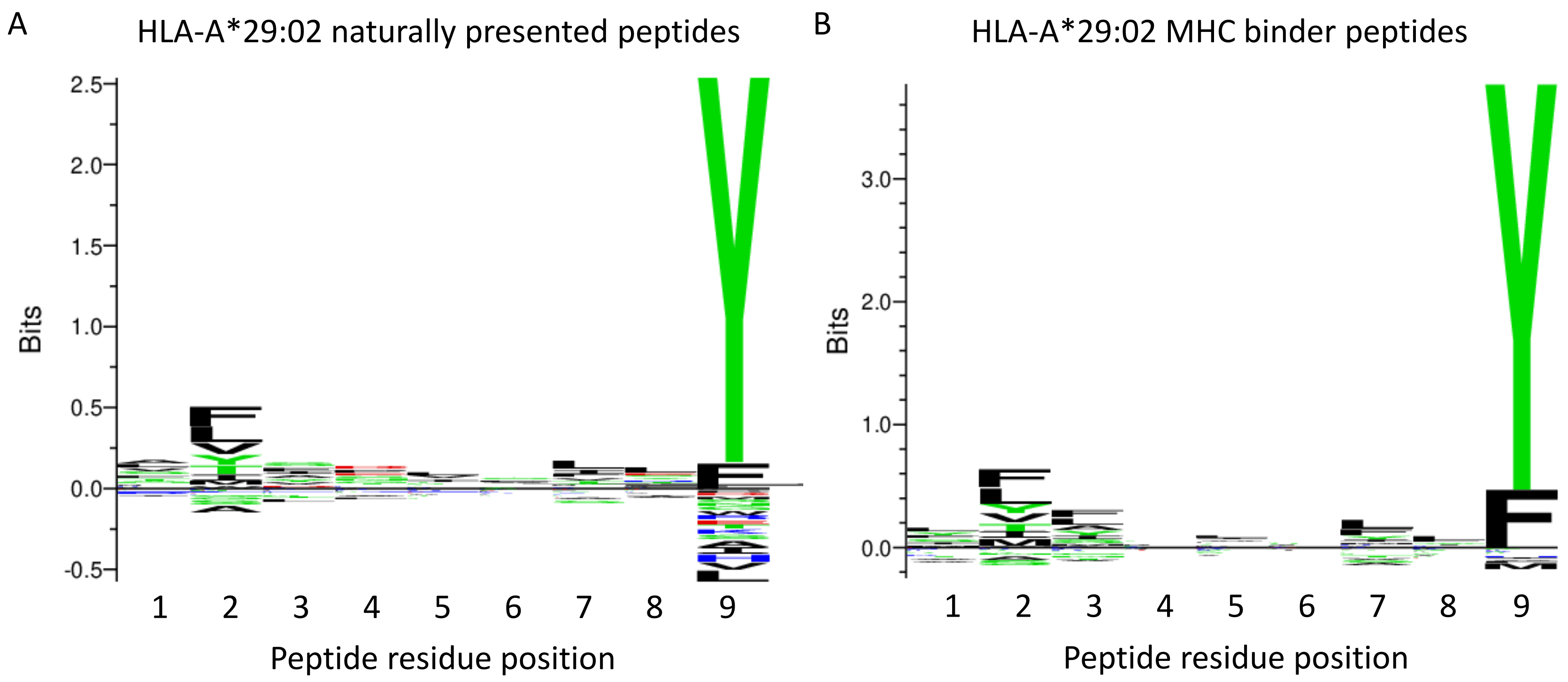

Supplementary Figure 6. HLA-A*29:02 peptide binding motif. The motif have been extracted from the netMHCpan4.1 web site reporting the (A) peptide binding motif from the naturally presented peptide and the (B) HLA-A*29:02 binder peptides (DOI: 10.1074/mcp.TIR119.001658).

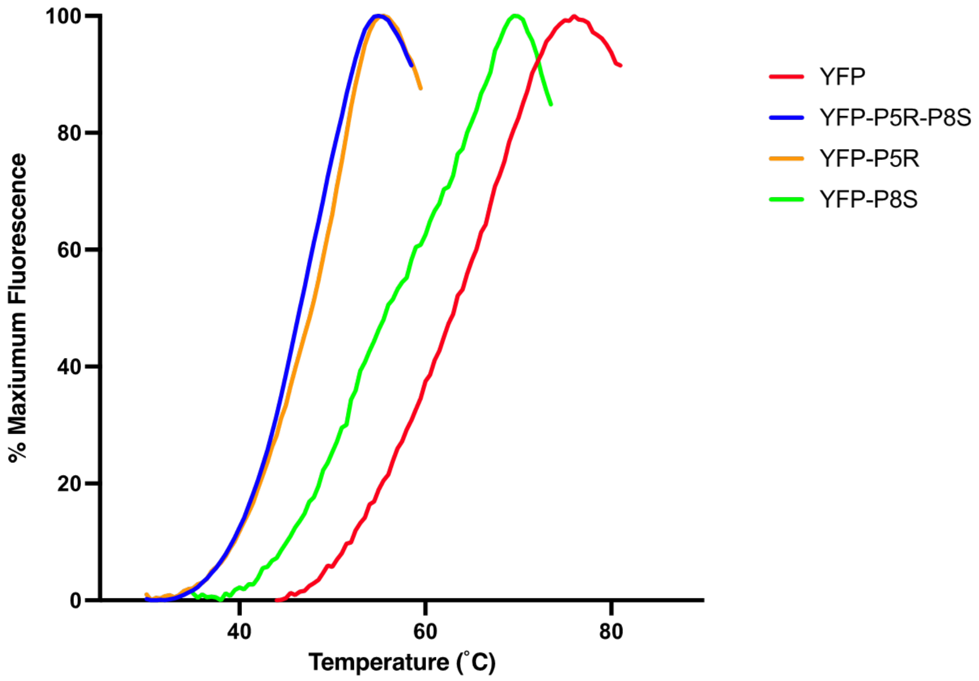

Supplementary Figure 7. Real-time fluorescent melting curves. The fluorescence signal was monitored continuously when the temperature was increased (1°C/min) and normalised to be plotted against the temperature to determine the thermal shift (Tm) of each pHLA complex represented in colour.

Supplementary Table 1: HLA types of COVID-19-convalescent study participants

Participant Code	Sex	Age	HLA Class I Alleles					
Q-014	F	61	A*03:01	A*29:02	B*07:02	B*44:03	C*07:02	C*16:01
Q-031	M	61	A*02:01	A*29:02	B*15:01	B*44:03	C*03:04	C*16:01
Q-043	M	56	A*01:01	A*29:02	B*08:01	B*44:03	C*07:01	C*16:01
Q-059	F	29	A*26:01	A*29:02	B*38:01	B*44:03	C*12:03	C*16:01
Q-001	M	20	A*03:01		B*07:02		C*07:02	
Q-002	F	53	A*02:01	A*32:01	B*15:01	B*44:02	C*03:03	C*05:01
Q-003	M	52	A*01:01	A*25:01	B*08:01	B*35:01	C*04:01	C*07:01
Q-004	F	30	A*02:01	A*03:01	B*07:02	B*44:02	C*05:01	C*07:02
Q-005	M	33	A*02:01	A*11:01	B*15:01	B*35:01	C*03:04	C*04:01
Q-006	M	48	A*02:01	A*24:02	B*07:02	B*18:01	C*07:01	C*07:02
Q-007	F	20	A*02:01	A*31:01	B*07:02	B*40:01	C*03:04	C*07:02
Q-008	F	48	A*02:01	A*03:01	B*07:02	B*44:02	C*07:02	C*07:04
Q-009	M	63	A*03:01	A*26:01	B*07:02	B*27:05	C*01:02	C*07:02
Q-010	M	63	A*02:01		B*15:01	B*44:03	C*03:03	C*16:01
Q-011	F	59	A*02:01		B*07:02	B*44:02	C*04:01	C*05:01
Q-012	F	71	A*02:01	A*03:01	B*44:02	B*51:01	C*01:02	C*05:01
Q-013	F	51	A*24:02	A*25:01	B*08:01		C*07:01	
Q-015	F	30	A*02:01		B*35:03		C*04:01	C*12:03
Q-016	F	25	A*03:01	A*24:03	B*35:01	B*38:01	C*04:01	C*12:03
Q-017	F	58	A*03:01	A*26:01	B*07:02		C*07:02	
Q-018	M	63	A*02:05	A*30:01	B*13:02	B*49:01	C*06:02	C*07:01
Q-019	F	60	A*01:01	A*24:02	B*08:01	B*27:05	C*01:02	C*07:01
Q-020	M	58	A*24:02	A*31:01	B*07:02	B*35:02	C*04:01	C*07:02
Q-021	F	26	A*01:01	A*24:02	B*08:01	B*35:02	C*04:01	C*07:01
Q-022	F	21	A*01:01	A*31:01	B*07:02	B*08:01	C*07:01	C*07:02
Q-024	F	56	A*01:01	A*03:01	B*07:02	B*08:01	C*07:01	C*07:02
Q-025	F	67	A*01:01	A*02:01	B*08:01	B*40:01	C*03:04	C*07:01
Q-026	F	39	A*01:01	A*30:02	B*40:01	B*49:01	C*03:04	C*07:01
Q-029	F	31	A*02:01	A*31:01	B*07:02	B*40:01	C*03:04	C*07:02
Q-032	F	25	A*02:01		B*27:05	B*44:02	C*02:151	C*05:01
Q-033	M	25	A*02:01	A*24:02	B*15:01	B*18:04	C*03:04	C*12:03
Q-035	M	62	A*02:01	A*03:01	B*07:02	B*08:01	C*07:01	C*07:02
Q-036	M	33	A*02:01	A*33:05	B*08:01	B*14:02	C*07:01	C*08:02
Q-037	F	26	A*02:01	A*03:01	B*15:01		C*03:04	
Q-038	F	41	A*01:01	A*02:01	B*08:01	B*44:02	C*05:01	C*07:01
Q-039	M	48	A*02:01		B*15:01		C*01:02	C*03:04
Q-040	F	74	A*02:01	A*32:01	B*44:02	B*44:03	C*04:01	C*05:01
Q-041	M	59	A*24:02	A*33:01	B*14:02	B*40:01	C*03:04	C*08:02
Q-042	M	75	A*02:01	A*03:01	B*13:02	B*40:01	C*03:04	C*06:02
Q-044	F	56	A*01:01	A*03:01	B*08:01	B*44:03	C*07:01	C*16:01
Q-045	F	65	A*02:01	A*03:01	B*07:02	B*14:02	C*07:02	C*08:02
Q-046	M	21	A*02:01	A*24:02	B*15:01	B*40:01	C*03:04	
Q-048	F	46	A*01:01	A*03:01	B*08:01	B*53:01	C*04:01	C*07:01
Q-049	M	51	A*01:01	A*03:01	B*07:02	B*56:01	C*01:02	C*07:02
Q-050	M	30	A*02:01		B*13:02	B*27:05	C*01:02	C*06:02
Q-051	F	68	A*02:13	A*32:01	B*44:02		C*05:01	
Q-052	M	67	A*03:01	A*24:02	B*07:02	B*44:03	C*07:02	C*16:01
Q-053	M	63	A*01:01	A*02:01	B*40:01	B*52:01	C*03:04	C*12:02
Q-054	F	64	A*02:01	A*68:01	B*27:05	B*51:01	C*01:02	C*15:02
Q-055	M	66	A*03:01	A*25:01	B*07:02	B*15:01	C*03:04	C*07:02
Q-056	M	23	A*01:01		B*37:01	B*57:01	C*06:02	
Q-057	M	33	A*02:01		B*08:01	B*27:05	C*01:02	C*07:01

Q-058	F	52	A*02:05	A*11:01	B*35:01	B*49:01	C*04:01	C*07:01
Q-060	F	29	A*01:01		B*08:01	B*15:01	C*01:02	C*07:01
Q-061	F	47	A*02:01	A*32:01	B*15:01	B*35:01	C*04:01	
Q-062	F	49	A*02:01		B*08:01	B*18:01	C*07:01	
Q-063	F	22	A*01:01	A*34:01	B*08:01	B*15:21	C*04:03	C*07:01
Q-064	F	36	A*01:01	A*03:01	B*08:01	B*35:01	C*04:01	C*07:01
Q-065	F	69	A*01:01	A*31:01	B*14:02	B*40:01	C*03:04	C*08:02

Supplementary Table 2: HLA types of vaccinated study participants

Participant Code (Sex, Age)	HLA Class I Alleles						Vaccine
GR049 (F, 42)	A*26:01	A*29:02	B*27:05	B*44:03	C*01:02	C*16.01	Vaxzevria
GR117 (M, 58)	A*29:02	A*31:01	B*44:03	B*51:01	C*15:02	C*16:01	Unknown
GR166 (M, 53)	A*01:01	A*29:02	B*08:01	B*44:03	C*07:01	C*16:01	Vaxzevria
GR123 (M, 43)	A*23:17	A*29:11	B*13:02	B*41:01	C*06:02	C*07:01	Unknown
GR001 (M, 43)	A*01:01	A*03:01	B*07:02	B*08:01	C*07:01	C*07:02	Vaxzevria
GR067 (F, 33)	A*03:01	A*03:01	B*07:02	B*07:02	C*07:02	C*07:02	Cominarty
GR127 (M, 55)	A*02:01	A*02:01	B*07:02	B*39:01	C*07:02	C*12:03	Vaxzevria
GR143 (F, 40)	A*02:01	A*02:20	B*07:02	B*44:03	C*07:02	C*16:01	Vaxzevria
GR170 (F, 53)	A*02:01	A*33:01	B*07:02	B*14:02	C*07:02	C*08:02	Vaxzevria

Supplementary Table 3: Published HLA-A*29:02 epitopes retrieved from IEDB

**Allele**	**Epitope**
HLA-A*29:02	CVADYSVLY
HLA-A*29:02	FVFKNIDGY
HLA-A*29:02	GAAAYYVGY
HLA-A*29:02	KVGGNYNYLY
HLA-A*29:02	LTDEMIAQY
HLA-A*29:02	NSFTRGVYY
HLA-A*29:02	SANNCTFEY
HLA-A*29:02	SFKEELDKY
HLA-A*29:02	SVASQSIIAY
HLA-A*29:02	SWMESEFRVY
HLA-A*29:02	VASQSIIAY
HLA-A*29:02	VFAQVKQIY
HLA-A*29:02	VGGNYNYLY
HLA-A*29:02	VLKGVKLHY
HLA-A*29:02	WFVTQRNFY
HLA-A*29:02	WTAGAAAYY

Supplementary Table 4: Data Collection and Refinement Statistics

**Data Collection Statistics**	**HLA-A*29:02-YFP**
Temperature	100K
Space group	P1
Cell Dimensions (a,b,c) (Å)	48.81, 64.47, 76.42, 106.53, 92.43, 97.51
Resolution (Å)	48.20 – 2.30 (2.38 – 2.30)
Total number of observations	75049 (7532)
Number of unique observations	37380 (3668)
Multiplicity	2 (2.1)
Data completeness (%)	95.4 (95.5)
I/σ_I	4.1(1.3)
R_{pim}[a] (%)	7.4(57.8)
CC½ (%)	99.3 (54.0)
**Refinement Statistics**	
Non-hydrogen atoms, Proteins	6344
Non-hydrogen atoms, Water	30
R_{factor}[b] (%)	19.2
R_{free}[b] (%)	23.8
Rms deviations from ideality	
Bond lengths (Å)	0.0086
Bond angles (°)	1.07
Ramachandran plot (%)	
Allowed region	100
Disallowed region	0
**PDB code**	7TLT

[a]$R_{p.i.m} = \Sigma_{hkl} [1/(N-1)]^{1/2} \Sigma_i \mid I_{hkl,i} - \langle I_{hkl} \rangle \mid / \Sigma_{hkl} \langle I_{hkl} \rangle$. [b] $R_{factor} = \Sigma_{hkl} \mid \mid F_o \mid - \mid F_c \mid \mid / \Sigma_{hkl} \mid F_o \mid$ for all data except ≈ 5% which were used for R_{free} calculation.

Supplementary Table 5: Thermal stability of the pHLA-A*29:02 complexes.

**Peptide name**	**Tm (°C) ± S.E.M.**
YFPLQSYGF	61.4 ± 3.3
YFPL**R**SY**S**F	46.6 ± 0.2
YFPL**R**SYGF	47.6 ± 0.3
YFPLQSY**S**F	56.9 ± 0.3

T_m represents the mean thermal midpoint temperatures from two independent experiments (n=2). S.E.M represents the standard error of the mean from these data.

Supplementary Table 6: Most common HLA-B*07:02 and HLA-A*02:01 epitope sequences in SARS-CoV-2 variants of concern.

	**Spike B*07:02**	**Spike A*02:01**
	SPRRARSVA	YLQPRTFLL
Alpha	-H-------	---------
Beta	---------	---------
Gamma	-H-------	---------
Delta	-R-------	---------
Omicron	-H-------	---------

Supplementary Table 7: HLA-A*29 distribution in worldwide populations (allelefrequencies.net, top 30 populations)

Line	Allele	Population	% of individuals who have the allele	Allele frequency (in decimals)	Sample size
1	A*29	Cameroon Bamileke 2		0.186	35
2	A*29	Spain Pas Valley		0.141	88
3	A*29	Spain Gipuzkoa Basque		0.13	100
4	A*29	South Africa Natal Zulu	24	0.125	100
5	A*29	South Africa Tswana	24.4	0.122	41
6	A*29	Norway pop 2		0.106	576
7	A*29	Mexico Queretaro Rural		0.1047	43
8	A*29	Burkina Faso Fulani		0.102	49
9	A*29	Mexico Veracruz, Orizaba		0.1	60
10	A*29	Mozambique		0.099	202
11	A*29	Spain Catalonia Girona		0.098	88
12	A*29	Spain Ibiza		0.098	88
13	A*29	Tunisia Ghannouch		0.091	82
14	A*29	Mexico Veracruz, Coatzacoalcos		0.0893	55
15	A*29	Saudi Arabia	16.7	0.0873	18
16	A*29	Madeira pop 2		0.087	173
17	A*29	Mexico Jalisco, Tonala		0.0857	35
18	A*29	Cameroon Bakola Pygmy	16	0.0834	50
19	A*29	Mexico Quintana Roo, Cancun		0.0833	48
20	A*29	Spain Murcia		0.083	173
21	A*29	Spain Sevilla	15.5	0.0827	278
22	A*29	Spain Minorca		0.082	94
23	A*29	Spain Arratia Valley Basque		0.08	83
24	A*29	Vietnam Hanoi		0.08	50
25	A*29	Argentina Corrientes	14.2	0.077	155
26	A*29	Spain North Cabuerniga		0.077	95
27	A*29	Spain, Castilla y Leon, Northwest,	13.8	0.0761	1,743
28	A*29	United Kingdom Orkney		0.0743	88
29	A*29	Venezuela Valencia and Maracaibo Caracus Mixed		0.073	96
30	A*29	Cuba Mixed pop 2		0.072	189

Supplementary Table 8: List of peptides and peptide pools used in this study

Name	Antigen	Description
PM-WCPV-APA3-1	ORF3A	Peptide Pool (15mers overlapping by 11)
PM-WCPV-NCAP-1	NCAP (N)	Peptide Pool (15mers overlapping by 11)
PM-WCPV-NS6-1	ORF6	Peptide Pool (15mers overlapping by 11)
PM-WCPV-NS7A-1	ORF7A	Peptide Pool (15mers overlapping by 11)
PM-WCPV-NS7B-1	ORF7B	Peptide Pool (15mers overlapping by 11)
PM-WCPV-NS8-1	ORF8	Peptide Pool (15mers overlapping by 11)
PM-WCPV-ORF9B-1	ORF9B	Peptide Pool (15mers overlapping by 11)
PM-WCPV-ORF10-1	ORF10	Peptide Pool (15mers overlapping by 11)
PM-WCPV-S-1	Spike (S)	Peptide Pool (15mers overlapping by 11)
PM-WCPV-VEMP-1	Envelope (E)	Peptide Pool (15mers overlapping by 11)
PM-WCPV-VME-1	Membrane (M)	Peptide Pool (15mers overlapping by 11)
PM-WCPV-Y14-1	Y14	Peptide Pool (15mers overlapping by 11)
EMPS-MCPV-S-1	Spike (S)	Matrix Pools
ORF3A-OP (Custom)	ORF3A	Matrix Pools

Supplementary Table 9: List of Reagents used in this study

Antibodies	Dilution	Source	Identifier/Clone
CD107a-FITC BD	1:10	BD Biosciences	Cat# 555800; H4A3
CD8-PerCP-Cy5.5	1:400	eBioscience	Cat# 45-0088-42; RPA-T8
CD4-Pacific Blue	1:200	BD Biosciences	Cat# 558116; RPA-T4
CD4-FITC	1:50	BD Biosciences	Cat#555346; RPA-T4
CD4-PE-Cy7	1:20	BD Biosciences	Cat# 560649; RPA-T4
Live/Dead Fixable Near-IR Dead Cell Stain	1:250	Life Technologies	Cat# L34975
IFN-g-AF700	1:50	BD Biosciences	Cat# 557995; B27
IFN-g-PE	1:200	BD Biosciences	Cat# 554701; B27
IL2-PE	1:50	BD Biosciences	Cat# 12- 7029-42, MQ1-17H12
TNF-APC	1:200	BD Biosciences	Cat# 554514; MAb11

Chemical and Peptides	Source	Identifier
RPMI-1640	Thermofisher	Cat# 21870092
penicillin/streptomycin	Life Technologies	Cat# 15140122
Foetal Bovine Serum	Life Technologies	Cat# 16000044
Interleukin-2	NIH	NA
GolgiPlug Protein Trnsp Inhb	BD Biosciences	Cat# 555029
Cytofix/Cytoperm W/Golgi Stop Kit	BD Biosciences	Cat# 554715
Paraformaldehyde (BD cytofix)	BD Biosciences	Cat# 554655
PBS	GIBCO	003002
Urea	Thermofisher	Cat# AJA817-5KG
L-Arginine	Sigma	Cat# A5131-1KG
Tris-HCl	Thermofisher	Cat# FSBBP152-5
EDTA	BDH	Cat# BDH9232-500G
glutathione reduced	Goldbio	Cat# G-155-500
glutathione oxidised	Goldbio	Cat# G-060-25
SYPRO orange	Thermofisher	Cat# S6650
Phenylmethylsulfonyl fluoride	Goldbio	Cat# P-470-25
NaCl	Thermofisher	Cat# CHE700/NACL-25KG
NH4SO4	Astral	Cat# BIOADB0060-500 g
Magnesium Chloride	Hampton Research	Cat# HR2-559
Bis-Tris propane	Hampton Research	Cat# HR2-103
Ethylene glycol	Sigma	Cat# 293237-1L
Acetonitrile	Honeywell	Cat# AH015-4A
Trifluoroacetic acid	Chem-supply	Cat# TS181-500M
Triisopropylsilane	Sigma-Aldrich	Cat# 233781-50G
Fmoc-O-tert-butyl-L-tyrosine	Chem-supply	Cat# 00495
Fmoc-L-leucine	Chem-supply	Cat#00145

N^{α}-Fmoc- N^{δ}-trityl-L-glutamine	Chem-supply	Cat# 02411
Fmoc-L-proline	Chem-supply	Cat# 02448
N^{α}-Fmoc-N^{ω}-(2,2,4,6,7-pentamethyldihydro-benzofuran-5-sulfonyl)-L-arginine	Chem-supply	Cat# 01964
Fmoc-O-tert-butyl-L-threonine	Chem-supply	Cat# 02460
Fmoc-L-phenylalanine	Chem-implex	Cat# 02443
Fmoc-L-valine	Chem-supply	Cat#02470
Fmoc-L-aspartic acid β-tert-butyl ester	Chem-supply	Cat# 00494
Fmoc-L-isoleucine	Chem-Implex	Cat# 02425
Fmoc-O-tert-butyl-L-serine	Chem-Implex	Cat# 02454
Fmoc-L-alanine	Chem-implex	Cat# 02369
Fmoc-Glycine	Chem-Implex	Cat# 02416
O-(1H-6-Chlorobenzotriazol-1-yl)-N,N,N',N'-tetramethyluronium hexafluorophosphate	Chem-Implex	Cat# 14260
1-Hydroxybenzotriazole hydrate	Sigma-Aldrich	Cat# 54802-50G-F
4-Benzyloxybenzyl alcohol resin	Chem-Implex	Cat# 01927
SARS-CoV-2 antigen overlapping peptide pools (ORF3A,NCAP,ORF6,ORF7A,ORF7B,ORF8,ORF9B, ORF10,Spike,Envelope, Membrane, Y14)	JPT	PM-WCPV-NCAP-1<br>PM-WCPV-NS6-1<br>PM-WCPV-NS7A-1<br>PM-WCPV-NS7B-1<br>PM-WCPV-NS8-1<br>PM-WCPV-ORF9B-1<br>PM-WCPV-ORF10-1<br>PM-WCPV-S-1<br>PM-WCPV-VEMP-1<br>PM-WCPV-VME-1<br>PM-WCPV-Y14-1
Spike overlapping peptide pools	Mimotopes	N/A
YFPLQSYGF, YFTSDYYQLY, YFPLRSYSF, CVADYSVLY, FVFKNIDGY, GAAAYYVGY, SANNCTFEY KVGGNYNYLY, LTDEMIAQY, NSFTRGVYY, SFKEELDKY, SVASQSIIAY, SWMESEFRVY, VASQSIIAY, VFAQVKQIY, VGGNYNYLY, VLKGVKLHY, WFVTQRNFY, WTAGAAAYY, SPRRARSVA, SHRRARSVA,SRRRARSVA, YLQPRTFLL	Genscript	N/A
YFPLRSYGF, YFPLQSYSF	La Trobe University	N/A

Software and algorithms	Source	Identifier
BD LSRFortessa FACSDiva software	BD Biosciences	N/A
AS-User GUI	Australian Synchrotron, ANSTO	N/A
XDS (Nov 1, 2016 BUILT=20161205)	XDS	N/A
PHASER (2.8.3)	CCP4	N/A

CCP4 suite (7.1.015)	CCP4	N/A
COOT (0.9.5)	Coot	N/A
BUSTER (2.10.3)	BUSTER	N/A
Pymol (1.7.4.5)	Schro¨ dinger	N/A
FlowJo software (Version 10.7.1)	FlowJo, LLC	N/A
GraphPad Prism 9 (version 9.3)	Graphpad	N/A

**Chromatography columns and Fluorimetry**	**Source**	**Identifier**
HiTrapQ column	GE Healthcare	17135101
ViiA 7 real-time PCR system	Applied Biosystems	N/A
RP-HPLC	Shimadzu	N/A
