# Supplementary material for: Ablation of CD8+ T cell recognition of an immunodominant epitope in SARS-CoV-2 Omicron variants BA.1, BA.2 and BA.3
Source: Nat Commun. 2022 Oct 27;13:6387. doi: 10.1038/s41467-022-34180-1 (PMC9607807; doi:10.1038/s41467-022-34180-1)
Supplement: Supplementary file 3 — Reporting Summary [file 41467_2022_34180_MOESM3_ESM.pdf]

nature portfolio

Corresponding author(s): Corey Smith
Manuscript Number : NCOMMS-22-03000A

Last updated by author(s): Sep 22, 2022

Reporting Summary

Nature Portfolio wishes to improve the reproducibility of the work that we publish. This form provides structure for consistency and transparency in reporting. For further information on Nature Portfolio policies, see our Editorial Policies and the Editorial Policy Checklist.

Statistics

For all statistical analyses, confirm that the following items are present in the figure legend, table legend, main text, or Methods section.

n/a	Confirmed	
☐	☒	The exact sample size (*n*) for each experimental group/condition, given as a discrete number and unit of measurement
☒	☐	A statement on whether measurements were taken from distinct samples or whether the same sample was measured repeatedly
☒	☐	The statistical test(s) used AND whether they are one- or two-sided<br>*Only common tests should be described solely by name; describe more complex techniques in the Methods section.*
☒	☐	A description of all covariates tested
☒	☐	A description of any assumptions or corrections, such as tests of normality and adjustment for multiple comparisons
☐	☒	A full description of the statistical parameters including central tendency (e.g. means) or other basic estimates (e.g. regression coefficient) AND variation (e.g. standard deviation) or associated estimates of uncertainty (e.g. confidence intervals)
☒	☐	For null hypothesis testing, the test statistic (e.g. *F*, *t*, *r*) with confidence intervals, effect sizes, degrees of freedom and *P* value noted<br>*Give P values as exact values whenever suitable.*
☒	☐	For Bayesian analysis, information on the choice of priors and Markov chain Monte Carlo settings
☒	☐	For hierarchical and complex designs, identification of the appropriate level for tests and full reporting of outcomes
☒	☐	Estimates of effect sizes (e.g. Cohen's *d*, Pearson's *r*), indicating how they were calculated

Our web collection on statistics for biologists contains articles on many of the points above.

Software and code

Policy information about availability of computer code

Data collection

Software and algorithms	Source	Identifier
BD LSRFortessa FACSDiva software (Version xxx)	BD Biosciences	N/A
MX2 Beamline	Australian Synchrotron, ANSTO	N/A
XDS	XDS	N/A
PHASER	CCP4	N/A
CCP4 suite	CCP4	N/A
COOT	Coot	N/A
BUSTER (Version 2.10)	BUSTER	N/A
Pymol	Schro¨ dinger	N/A

Data analysis

Software	Source	Identifier
FlowJo software (TreeStar)	FlowJo, LLC	N/A
GraphPad Prism 9 (version 9.3)	Graphpad	N/A

For manuscripts utilizing custom algorithms or software that are central to the research but not yet described in published literature, software must be made available to editors and reviewers. We strongly encourage code deposition in a community repository (e.g. GitHub). See the Nature Portfolio guidelines for submitting code & software for further information.

Data

Policy information about availability of data

All manuscripts must include a data availability statement. This statement should provide the following information, where applicable:

- Accession codes, unique identifiers, or web links for publicly available datasets
- A description of any restrictions on data availability
- For clinical datasets or third party data, please ensure that the statement adheres to our policy

Source	Accession Id
Protein Data Bank	
https://www.rcsb.org/	7TLT
GISAID	
https://gisaid.org/	BA.1-EPI_ISL_9049930
	BA.2-EPI_ISL_13059703
	BA.3-EPI_ISL_12650713
	BA.4-EPI_ISL_14858758
	BA.5- EPI_ISL_12780920

Human research participants

Policy information about studies involving human research participants and Sex and Gender in Research.

Reporting on sex and gender	We recruited two cohorts: COVID-19-convalescent participants: COVID-19-recovered donors were over the age of 18, had been clinically diagnosed by PCR with SARSCoV-2 infection, and had subsequently been released from isolation following resolution of symptomatic infection. A total of 58 participants were recruited in May and June 2020 from the south-east region of Queensland, Australia. The majority of participants were returned overseas travellers. Participants ranged in age from 20 to 75, 24 were male and 34 were female, and were a median of 62 (46 – 124) days post-initial diagnosis. Blood samples were collected from all participants to isolate peripheral blood mononuclear cells (PBMCs) to assess SARS-CoV-2 immunity. Healthy donors over the age of 18, with no known COVID-19 infection or exposure, were recruited. These donors are referred to as unexposed throughout the manuscript. A total of 9 unexposed donors were recruited, ranging in age from 19 to 56 (average of 33 years old), 5 were male, 4 were female. Informed consent was obtained from all participants. The HLA typing was performed by AlloSeq Tx17 (CareDx Pty Ltd, Fremantle, Australia), or Australian Red Cross Victorian Transplant and Immunogenetics Service (Melbourne, Australia), or PathWest Laboratory Medicine, Fiona Stanley Hospital using AllType NGS high resolution typing on the IonTorrent NGS platform, and these details are provided In Supplementary Table 1 and 2.
Population characteristics	All participants were over the age of 18 and provided informed consent to participate in this study. COVID-19-convalescent individuals donated blood for PBMC isolation following resolution of symptoms (n=59), while vaccinated individuals donated blood 28 days after their second vaccine dose (n=9). HLA typing was performed on each donated sample. HLA typing is provided in supplementary tables in the manuscript.
Recruitment	Both recovered covid-19 and vaccinated volunteers donated their blood at QIMR Berghofer after written consent was obtained. The vaccinated participants were given either an adenovirus vector (Vaxzevria) or an mRNA (Cominarty) vaccine.
Ethics oversight	This study was approved by the QIMR Berghofer Medical Research Institute Human Research Ethics Committee and was performed according to the principles of the Declaration of Helsinki.

Note that full information on the approval of the study protocol must also be provided in the manuscript.

Field-specific reporting

Please select the one below that is the best fit for your research. If you are not sure, read the appropriate sections before making your selection.

☒ Life sciences ☐ Behavioural & social sciences ☐ Ecological, evolutionary & environmental sciences

For a reference copy of the document with all sections, see nature.com/documents/nr-reporting-summary-flat.pdf

Life sciences study design

All studies must disclose on these points even when the disclosure is negative.

Sample size	Sample size calculation was not performed. It is determined by the availability of samples.

Data exclusions	No data were excluded
Replication	Due to the low sample available for the human PBMC samples used in the study, each assay was performed from each individual a single time and could not be replicated. To demonstrate the reproducibility of our findings we used a number of follow-up assay to demonstrate that the immunodominance and cross-reactive patterns seen in our T cell responses were reproducible and used multiple donors with the same HLA-type. We also performed follow-up in vitro culture analysis on the vaccinated individuals in our cohort to validate the responses we observed directly ex vivo. We confirm all the attempts at validation were successful.
Randomization	Recovered COVID-19 and vaccinated donors were not randomized since we include all volunteers and selected based on HLA-A*29:02 positive or negative
Blinding	Experiments were not blinded as all participants received the vaccine and the study is observational

Reporting for specific materials, systems and methods

We require information from authors about some types of materials, experimental systems and methods used in many studies. Here, indicate whether each material, system or method listed is relevant to your study. If you are not sure if a list item applies to your research, read the appropriate section before selecting a response.

Materials & experimental systems

n/a	Involved in the study
☐	☒ Antibodies
☒	☐ Eukaryotic cell lines
☒	☐ Palaeontology and archaeology
☒	☐ Animals and other organisms
☒	☐ Clinical data
☐	☒ Dual use research of concern

Methods

n/a	Involved in the study
☒	☐ ChIP-seq
☐	☒ Flow cytometry
☒	☐ MRI-based neuroimaging

Antibodies

Antibodies used

Antibodies	Source	Identifier
CD107a-FITC BD	Biosciences/eBioscience	Cat# 555800; RRID:AB_396134
CD8-PerCP-Cy5.5	BD Biosciences/eBioscience	Cat# 565310; RRID:AB_2687497
CD4-Pacific Blue	BD Biosciences	Cat# 558116; AB_397037
CD4-PE-Cy7	BD Biosciences	Cat# 560649; RRID:AB_1727475
Live/Dead Fixable Near-IR Dead Cell Stain	Life Technologies	Cat# L34975
IFN-g-AF700	BD Biosciences	Cat# 557995; RRID:AB_39697
IFN-g-PE	BD Biosciences	Cat# 554701; RRID:AB_395518
IL2-PE	Thermo Fisher/ Invitrogen	Cat# 12- 7029-42, RRID: AB_2572651
TNF-APC	BD Biosciences	Cat# 554514; RRID:AB_398566

Validation

Antibodies used in this study have undergone quality control testing as outlined by the supplier and were used for their intended purpose

Dual use research of concern

Policy information about dual use research of concern

Hazards

Could the accidental, deliberate or reckless misuse of agents or technologies generated in the work, or the application of information presented in the manuscript, pose a threat to:

No	Yes	
☒	☐	Public health
☒	☐	National security
☒	☐	Crops and/or livestock
☒	☐	Ecosystems
☒	☐	Any other significant area

Experiments of concern

Does the work involve any of these experiments of concern:

No	Yes	
☒	☐	Demonstrate how to render a vaccine ineffective
☒	☐	Confer resistance to therapeutically useful antibiotics or antiviral agents
☒	☐	Enhance the virulence of a pathogen or render a nonpathogen virulent
☒	☐	Increase transmissibility of a pathogen
☒	☐	Alter the host range of a pathogen
☒	☐	Enable evasion of diagnostic/detection modalities
☒	☐	Enable the weaponization of a biological agent or toxin
☒	☐	Any other potentially harmful combination of experiments and agents

Flow Cytometry

Plots

Confirm that:

☒ The axis labels state the marker and fluorochrome used (e.g. CD4-FITC).

☒ The axis scales are clearly visible. Include numbers along axes only for bottom left plot of group (a 'group' is an analysis of identical markers).

☒ All plots are contour plots with outliers or pseudocolor plots.

☒ A numerical value for number of cells or percentage (with statistics) is provided.

Methodology

Sample preparation	Cultured T cells (5 × 105 per test) or PBMC (2 × 106 per test) were stimulated separately with the SARS-CoV-2 overlapping peptide pools (1 µg/mL of each peptide), individual defined epitopes (1 µg/mL) or with a cytokine stimulation cocktail (eBioscience), and incubated for 4 hours (T cells) or 6 hours (PBMC) at 37°C in the presence of GolgiPlug, GolgiStop and anti-CD107a-FITC (BD Biosciences). Following stimulation, cells were washed and stained with anti-CD8-PerCP-Cy5.5 (eBioscience), anti-CD4-Pacific Blue or anti-CD4-PE-Cy7 (BD Biosciences) and live/dead fixable near-IR dead cell stain (Life Technologies) for 30 minutes at 4°C before being fixed and permeabilized with Fixation/Permeabilization solution (BD Biosciences). After 20 minutes of fixation, cells were washed in BD Perm/Wash buffer (BD Biosciences) and stained with anti-IFN-g-Alexa Fluor 700 or anti-IFNg-PE (BD Biosciences), anti-IL-2-PE (ThermoFisher/ Invitrogen) and anti-TNF-APC (BD Biosciences) for a further 30 minutes at 4°C. Finally, cells were washed again and acquired using a BD LSRFortessa with FACSDiva software
Instrument	BD LSRFortessa FACSDiva - BD biosciences
Software	FlowJo software (TreeStar), FlowJo, LLC
Cell population abundance	*Describe the abundance of the relevant cell populations within post-sort fractions, providing details on the purity of the samples and how it was determined.*
Gating strategy	Flow cytometer gating strategy for T cells.<br>Lymphocytes were defined by SSC-A and FSC-A. Single cell population from lymphocytes were defined using FSC-H/FSC-A. Viable cells from single cells were defined by gating FSC-A/ live/dead fixable near-IR dead cell stain (Life Technologies) . CD8

population from viable cells were gated using anti-CD8-PerCP-Cy5.5(eBioscience) and anti-CD4-Pacific Blue or anti-CD4-PE-Cy7 (BD Biosciences). From anti-CD8-PerCP-Cy5.5 , we represent Representative IFN-g production by ORF3A and spike-specific CD8+ T cells were obtained. The gating strategy is shown in the Supplementary Figure 1a and 1b

☒ Tick this box to confirm that a figure exemplifying the gating strategy is provided in the Supplementary Information.
